# Bacteriophages in the Dairy Environment: From Enemies to Allies

**DOI:** 10.3390/antibiotics6040027

**Published:** 2017-11-08

**Authors:** Lucía Fernández, Susana Escobedo, Diana Gutiérrez, Silvia Portilla, Beatriz Martínez, Pilar García, Ana Rodríguez

**Affiliations:** Instituto de Productos Lácteos de Asturias (IPLA-CSIC), Paseo Río Linares s/n, Villaviciosa, 33300 Asturias, Spain; s.escobedo@ipla.csic.es (S.E.); dianagufer@ipla.csic.es (D.G.); silvia.portilla@ipla.csic.es (S.P.); bmf1@ipla.csic.es (B.M.); pgarcia@ipla.csic.es (P.G.); anarguez@ipla.csic.es (A.R.)

**Keywords:** bacteriophages, dairy industry, pathogens, lactic acid bacteria, fermentation failure, biofilms, antimicrobial resistance

## Abstract

The history of dairy farming goes back thousands of years, evolving from a traditional small-scale production to the industrialized manufacturing of fermented dairy products. Commercialization of milk and its derived products has been very important not only as a source of nourishment but also as an economic resource. However, the dairy industry has encountered several problems that have to be overcome to ensure the quality and safety of the final products, as well as to avoid economic losses. Within this context, it is interesting to highlight the role played by bacteriophages, or phages, viruses that infect bacteria. Indeed, bacteriophages were originally regarded as a nuisance, being responsible for fermentation failure and economic losses when infecting lactic acid bacteria, but are now considered promising antimicrobials to fight milk-borne pathogens without contributing to the increase in antibiotic resistance.

## 1. Introduction

### 1.1. Origins and Industrialization of Dairy Production

Archaeological evidence indicates that already in ancient times, the people of Mesopotamia learned to domesticate milk-producing animals, using and preserving milk for nourishment [1]. Thousands of years later, milk is still the most consumed dairy product worldwide, playing a fundamental role in the diet of all populations [2,3]. It is precisely from the exercise of milk extraction by man that the dairy industry was developed [1,4]. Indeed, cheese and yogurt, the first dairy derivatives, were accidentally discovered as a result of the difficulties encountered to transport and preserve milk. From that time to the present, there has been a continuous development of new and improved dairy products. One of the most striking features of the traditional dairy industry is the manner in which chemical, microbiological, physical, and engineering principles were integrated to allow the manufacture of high quality and safe products. This multidisciplinary strategy has led to the wide variety of products available today. Nowadays, aspects like the availability and presentation of products are very important for the consumer. An example of this is the diversification of dairy products by the inclusion of fruits and cereals [3,5,6]. Moreover, the creation of new and sophisticated products that contribute to improving the health of final users, the so-called functional foods, is on high demand [2,5]. Some examples include products with added vitamins and minerals or those supplemented with living beneficial microorganisms (probiotics). Besides dairy products, the technological development of the dairy industry has made it possible to separate solids from milk, and subsequently transform these components into raw material for other food industry sectors [4,7]. It is also worth noting that the diversity of dairy products varies considerably from region to region depending on dietary habits, available milk-making technologies, market demand, and sociocultural circumstances [8].

### 1.2. Economic Importance of the Dairy Industry in Different Countries

The dairy sector is a dynamic global industry that plays an important economic role in the agricultural sector of most industrialized and developing countries [8,9]. Currently, in the face of rising global demand and imminent industrial globalization, there has been an increase in both the scope and the intensity of world trade of dairy products [8]. Based on data estimates by the Food and Agricultural Organization (FAO), world milk production for 2016 was 817 million tons. In addition, the expected increase in global demand and production of dairy products until 2025 is estimated to be around 6–20 percent [9]. The most important milk producers are Europe, Asia and the Americas. More specifically, the European Union (EU) is the largest producing economic region worldwide, while India is the largest producer as a country [10]. According to the International Dairy Federation [9], milk production has increased by 50 percent in the last three decades, with a total of 150 million smallholders around the world participating in this activity. On the other hand, developed countries account for one-third of the world milk production, while the remaining two-thirds correspond to developing countries. In developing countries, however, growth in the dairy sector is limited by refrigeration, marketing and transportation problems as well as nutritional and zootechnical issues [8]. Thus, smallholders often lack the necessary skills to manage their farms as companies because they have limited access to animal health services, genetic improvement and training of personnel, which results in low yields and poor quality milk. In addition, the economic importance of dairy production both nationally and internationally is directly related to the sustainability of pasture production areas and the size of herds [11]. Other important factors that influence the success of the dairy sector are the degree of government intervention through subsidies and the demand in the export markets. Furthermore, the success of dairy development programs in different countries also depends to a large extent on traditional habits of consumption of dairy products [7,10]. Nonetheless, food safety remains a key global challenge in the dairy industry of any country to prevent economic losses and health concerns. Within this context, bacteriophages (or phages) have consistently played a significant role in the success of the dairy industry. Indeed, bacterial fermentation processes are threatened by contamination of raw milk with phages that infect lactic acid bacteria. This makes necessary the development of techniques to ensure control of the phage load in starting materials and equipment. In contrast, more recently, phages have been proposed as biocontrol agents to eliminate pathogenic or spoilage bacteria in dairy products. This review aims to summarize and discuss both the negative and positive impact of phages in dairy settings, depending on their specific bacterial hosts.

## 2. Bacteriophages as Unwanted Guests

**Figure 1:**
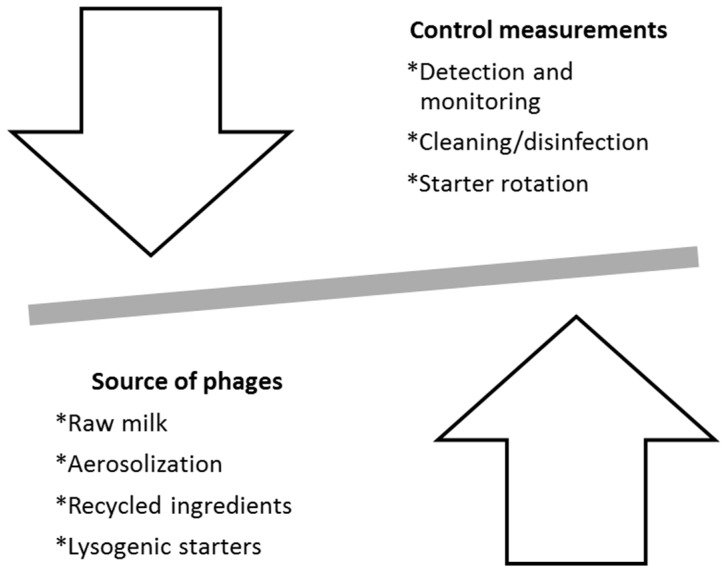
Factors that contribute to the presence of phages in dairy settings.

Phage infection of dairy starter cultures remains the main cause of fermentation failures in the dairy industry. Phage outbreaks can lead to substantial economic losses due to manufacturing delays, waste of ingredients, lower quality product, growth of spoilage and pathogenic microorganisms or even total production loss [12]. Close monitoring of entry routes, quick and effective phage detection methods and control measurements are currently applied to reduce the risk of phage propagation within dairy settings (Figure 1).

### 2.1. Sources of Contamination

The sources of phage entry into dairy plant facilities and dissemination routes must be identified in order to implement corrective actions to limit their propagation. Due to the wide diversity of phages present in raw milk, either as free virions or as prophages in wild lactic acid bacteria (LAB) strains, milk is considered to be the primary entry route for phages into the dairy environment [13]. As much as 10% of milk samples obtained from different dairies in Spain yielded viable *Lactococcus lactis* phages, while lactococcal and streptococcal phages were detected in 37% of raw milk samples used for yogurt production [14,15].

Personnel and equipment movement, raw materials handling, air displacements around contaminated surfaces and liquid splashes can aerosolize viruses and cause dissemination of phage particles in the air to the entire factory environment [16]. Concentrations ranging from 10^2^ PFU/m^3^ to 10^8^ PFU/m^3^ in air have been detected in different areas of a cheese manufacturing plant during the fermentation process [17]. A variety of samplers are now available for viral detection in the air; however, there is no standard sampling procedure [18]. In many cases, these devices may have damaging effects on the virus structure that can lead to false-negative results; that is why analytical methods that are independent of viral infectivity, such as quantitative PCR (qPCR), are more suitable for the analysis of air samples [19]. Other reservoirs of phages include materials and equipment used in the manufacturing process as well as surfaces in dairy facilities. Phages can be found in places where conditions for development of their host are favorable and where cleaning and disinfection are difficult.

A common practice in the manufacturing of yogurt and other fermented products consists in the utilization of reconstituted milk from powder and whey proteins obtained from cheese production to increase the product yield and improve the texture and nutritional value of the final products [20]. However, whey proteins may protect phages during heat; there is a correlation between thermal stability of molecular structures and their ability to protect lactococcal virulent phage P1532 from thermal treatments [21]. In addition, whey protein concentrate often contains high temperature-resistant phages, which are able to survive pasteurization and contaminate starters during the manufacturing process [14]. Furthermore, separation and concentration steps of the whey products, consisting in ultrafiltration and microfiltration, may also increase significantly phage titers in these ingredients [22].

LAB strains used as starter cultures can also be a source of phages since they may contain temperate phages integrated into the bacterial chromosome. Lysogeny is widely distributed among dairy lactococci, lactobacilli and with lower incidence in *Streptococcus thermophilus* strains [23,24]. Prophages may be induced and enter into the lytic life cycle under stress conditions such as heat, salts, bacteriocins, starvation, ultraviolet light or may also occur naturally with a frequency of even up to 9% [25,26,27].

### 2.2. Detection and Elimination

Great research efforts have focused on early detection of infective phages in dairy manufacturing. Phage monitoring methods include microbiological and molecular assays designed for rapid, low cost and high sensitive evaluation [28].

One of the most common methods for the detection of phages from industrial dairy plants is the activity test based on the acidification rate of milk that provides a reliable indication of their presence when acid production slows down. Acidification can be evaluated by pH measurements, color change of an indicator compound or variations in the electrical conductance of milk [29]. Another method is the double layer plaque assay, which allows a quantitative analysis of infective phage levels, but requires availability of a sensitive strain [30,31]. Flow cytometry can also be used for detection lysed bacterial cells that are found late in the lytic cycle, allowing an accurate and rapid monitoring of phage contamination [32].

Because microbiological tests are time consuming and mostly rely on the availability of single indicator strains, a number of alternative molecular methods focused on detecting the presence of phage particles or their components (DNA, proteins) have been developed. Immunological assays are based on the use of specific antibodies against principal structural proteins of the virion, while viral DNA can be detected with specific DNA-hybridization probes or by polymerase chain reaction [28]. PCR methods have been successfully adapted to detect and identify phages in different stages of dairy product manufacture. In a single reaction, multiplex PCR test allows the detection of several of the most common phages infecting LAB, such as *L. lactis* phage species P335, 936, and c2 and phages infecting *S. thermophilus* and *Lactobacillus delbrueckii* [15,33]. More sensitive than conventional PCR, real-time qPCR can be used to estimate the copy number of a target gene, allowing quantitative viral contamination diagnosis. By using different fluorogenic reporters in the same reaction it is possible to develop multiplex qPCR to detect different targets [34]. qPCR suppliers constantly offer new solutions to get automated systems adapted to industrial needs. Recently, phage metagenomics studies have been conducted to assess the biodiversity and dynamics of phage populations in dairy settings, providing a rational basis for suitable control strategies [35].

### 2.3. Control Methods

Significant progress in the control of phage populations within the dairy sector has been made in order to keep these bacterial viruses at bay. Although cleaning of equipment and facilities can remove a large proportion of microorganisms, the presence of residual LAB may increase the risk of phage contamination. The role of disinfection is to kill microorganisms that survived the cleaning procedures, reducing the spread of phages within the facility. Disinfectants active against bacteria are not always efficient to inactivate phages [36]. Several biocides used in the dairy industry as well as cleaning procedures have been tested for viral effectiveness on different phages infecting LAB strains. Peracetic acid and sodium hypochlorite containing products are shown to be the most efficient biocides for inactivation of phage particles, while ethanol and isopropanol were usually not effective [37]. The majority of disinfectants consist of several biocides and they must ensure the lack of negative impact on the final product and be able to degrade into harmless final compounds. Combining biocides and heat or using them at extreme pH conditions have shown to give the best results [38]. Photocatalysis intended to destroy fungi, bacteria and spores in the air has been recently explored for inactivating viruses infecting *Lactobacillus casei*, *Lb. delbrueckii* and *Lactobacillus plantarum* [39]. Photocatalytic reaction has shown to completely eliminate two 936-type phages, CHD and QF9 within 120 and 60 min of exposure; respectively [39]. Of note, UV-A radiation assayed by the authors has the advantage of safe use, thus allowing their application for long periods even in the presence of personnel.

The viral load of the ingredients used in dairy production should be reduced as much as possible. Although heating can reduce the activity of phage particles, many LAB phages are not inactivated by classical pasteurization procedures (63 °C for 30 min or 72 °C for 15 s). Therefore, emerging non-thermal technologies such as pulsed electric field, high hydrostatic pressure and high pressure homogenization as well as the combination with heat are currently being explored for inactivating phages [40]. It is important to take into consideration that phages also react differently to heat depending on the medium. Moreover, protective effects due to the presence of proteins, salt or fat have been reported [21,22].

Phage inhibitory media have been developed for starter propagation in dairy plants. The addition of components that inhibit or delay phage propagation such as chelating agents, sodium tripolyphosphate or purified phage peptides can help protect from further phage infection [41,42,43].

Rotation of defined phage-free cultures is an efficient phage control method to avoid recontamination by the same phage and the build-up of specific phages. A follow up is necessary in order to detect the emergence of new virulent phages to adjust the strain rotation protocol. Recently, a multiplex PCR method based on the genetic locus of the cell wall polysaccharide that acts as phage receptor for many lactococcal phages has been developed to predict phage susceptibility and aid to design suitable starter rotation schemes [44].

The availability of alternative phage resistant starters is of paramount importance and many efforts are being made to search for potential new starter bacteria with different phage sensitivity profiles or to engineer phage-resistant starters. Bacteria have developed natural defense mechanisms against phage infection based on adsorption inhibition, blockage of phage DNA injection, restriction-modification, abortive infection and CRISPR-Cas systems [45]. Many of these systems are plasmid encoded and can be moved from one strain to another for genetically improving dairy starters. Isolation of spontaneous bacteriophage insensitive mutants (BIMs) is a feasible alternative for bacteria without conjugative plasmids, and involves no genetic manipulation. On the other hand, construction of genetically engineered strains has been intensively studied. Several genetic tools, based in the LAB native phage defense mechanisms as well as phage elements have been designed. Examples of these engineered antiphage approaches include cloning of replication origin, antisense RNA technology, phage triggered suicide systems, overproduction of phage proteins, DARPins and neutralizing antibody fragments [12]. Nevertheless, legislation and consumers’ concerns regarding genetically modified organisms (GMOs) makes its application to dairy industry difficult.

## 3. Problems Associated with Bacterial Contamination

### 3.1. Foodborne Infectious Diseases in Dairy Products

Ensuring access to safe food products remains one of the major global health challenges. Indeed, foodborne diseases constitute a sanitary and economic burden in countries all over the world. To be effective, food safety measures require the participation of all the different actors along the food supply chain, “from farm to fork”, including farmers, manufacturers, vendors and consumers. This has become particularly difficult in our global market economy, as these different actors are often far away from each other, frequently across national borders. In this context, adequate regulatory frameworks need to be in place to ensure that the required safety standards are met throughout the process. Nonetheless, foodborne infections are still a major health care concern, with a total of 600 million people falling ill and 420,000 dying every year from eating contaminated food [46].

**Figure 2:**
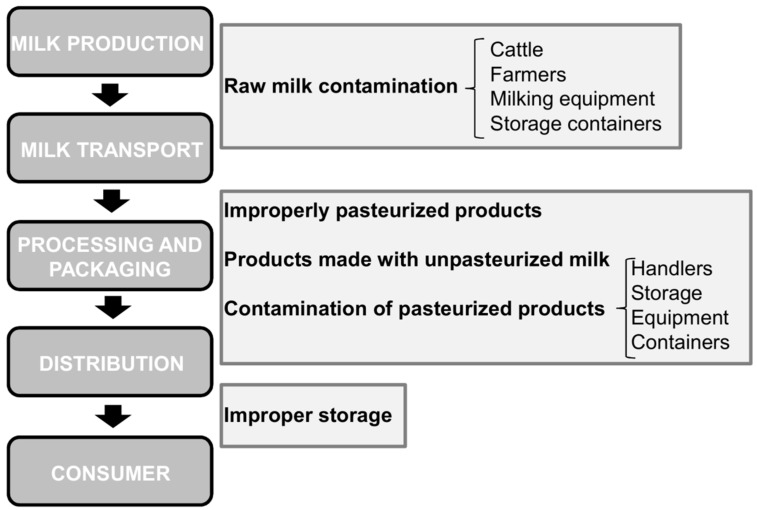
Schematic representation of different points of the dairy supply chain susceptible to microbial contamination.

Dairy products can get contaminated at different points along the production chain (Figure 2). For instance, raw milk can carry microorganisms from the udder or teat canal, the milking equipment, storage containers, the animal’s or handler’s skin, etc. [47]. Since some of these microbes can be human pathogens, milk can be a potential source of infections if consumed unpasteurized. These pathogens may even persist in aged products made from raw milk, like some traditionally-manufactured cheeses [48]. Pasteurization, on the other hand, can kill most potentially dangerous microorganisms present in milk [47]. However, outbreaks may still occur due to improper pasteurization or post-pasteurization contamination of the milk. Indeed, proper cleaning and hygiene procedures are essential to prevent milk-borne infections.

The pathogens commonly found in the dairy environment include viruses, parasites, fungi and bacteria [49]. Some of the most notorious bacterial pathogens are *Brucella* spp., *Campylobacter jejuni*, *Bacillus cereus*, Shiga toxin-producing *Escherichia coli* (*E. coli* O157:H7), *Staphylococcus aureus*, *Listeria monocytogenes*, *Coxiella burnietti*, *Mycobacterium tuberculosis*, *Mycobacterium bovis*, *Salmonella* spp. and *Yersinia enterocolitica*. Consumption of unpasteurized milk and its derived products is the main source of contamination for most of these pathogens [50,51,52,53,54,55,56,57,58,59,60,61,62]. Although unpasteurized milk is not easily available to consumers, it is still consumed by dairy farmers and raw-milk health advocates [51,63]. The human pathogenic bacterium *S. aureus* is one of the microorganisms responsible for mastitis in dairy cows and can also be a source of raw milk contamination [64]. However, this microbe can frequently contaminate food after pasteurization as a result of improper handling during production. *S. aureus* is also problematic due to the production by some strains of heat stable enterotoxins that cannot be easily destroyed by cooking the product [65]. As a result, contaminated products will remain dangerous even after the bacterium has been killed, potentially leading to intoxications.

Taking all of this into account, it is evident that proper hygiene and disinfection measures are essential along the dairy production chain, from the handling of dairy cows to the final product before it reaches the consumer. On top of that, consumers need to be aware that following the instructions for preservation of dairy products and obeying expiry dates are important to ensure their safety.

### 3.2. Antimicrobial Resistance in the Dairy Environment

Antimicrobials have been overused and misused in human and veterinary medicine ever since their introduction in the clinic. One of the main consequences of this has been the spread of antibiotic resistance determinants amongst microorganisms, including human pathogens, even in environments where antimicrobials themselves were not present [66]. This increase in antibiotic resistance has ultimately led to a decrease in the efficacy of routine disinfection regimes. Indeed, strains belonging to some species have acquired resistance to almost all antibiotics available in the market. The so-called “superbugs” have raised the alarms within the medical and scientific community at large as an indicator that the antibiotic era might be coming to an end. From a less dramatic perspective, perhaps superbugs remind us of the need to understand resistance mechanisms and develop new antimicrobials.

The use of antibiotics in the context of the dairy industry is subject to strict regulations, which are in place to avoid the presence of antibiotic residues in milk aimed for human consumption. For instance, in the US, safety standards for milk are specified in the Grade “A” Pasteurized Milk Ordinance and the Regulation EC 853/2004 defines food safety standards for foodstuffs in the EU [67,68]. In the dairy environment, antimicrobials are used for the treatment of infections in cattle, as growth promoters and as prophylactic agents. The most prevalent infectious illness affecting dairy cattle is mastitis, followed by respiratory infections, lameness, infections of the reproductive system and diarrhea/gastrointestinal tract infections [69]. In many cases, cows require antibiotic treatment with cephalosporins and tetracycline being the most frequently used for mastitis and lameness, respectively [69]. Also, farmers often administer antibiotics to prevent infections, usually penicillin G or dihydrostreptomycin, following the end of the lactation period, the so-called dry cow therapy [69]. The most common routes of antibiotic administration in cows are intramammary and intramuscular [70].

Generalized used of antimicrobials in agriculture and animal farming is considered a potential risk factor for the increased prevalence of antimicrobial resistance in bacteria from food-producing animals [71,72]. Thus, antibiotic pressure would favor the selection and spread of resistance markers by horizontal transfer [73,74,75]. It must be pointed out, however, that there is no definitive scientific evidence of a direct link between the two. Nevertheless, there have been numerous studies that tried to determine whether antibiotic resistance increased in microorganisms from dairy environments as a result of antimicrobial exposure. However, the results obtained have shown contradictory information. Thus, some studies point that there is an increase in antibiotic resistance over time under antibiotic pressure, while others show no change whatsoever, with differences observed for certain species or antimicrobials [76,77]. Also, some studies have assessed whether there are differences in the amount of antimicrobial resistant organisms in conventional versus organic (antibiotic-free) dairies. For instance, Pol and Ruegg [78] observed that some microorganism-antibiotic combinations were indeed dependent on the farm type while others showed no difference.

Due to the concern regarding antibiotic resistance in pathogenic bacteria, there has been a boom in research regarding the development of novel antimicrobials and new disinfection regimes. Amongst these therapeutic alternatives, phages have been gaining particular attention, as we will discuss below.

## 4. Bacteriophages as Unexpected Allies

### 4.1. Phages as Disinfectants and Preservatives in the Dairy Industry

**Figure 3:**
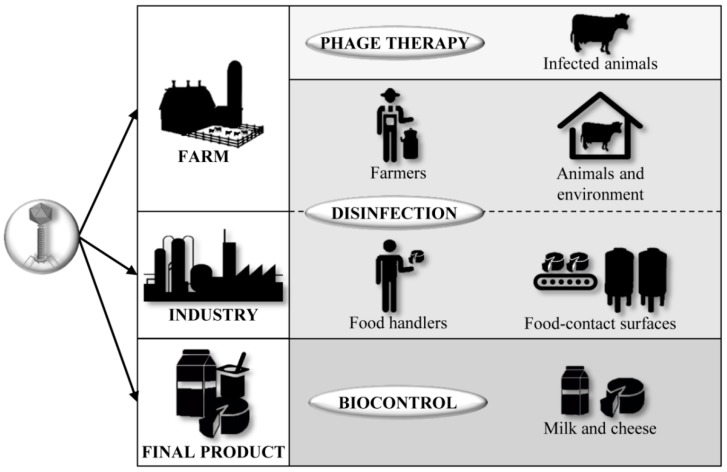
Principal points of disinfection and biocontrol along the dairy chain (from “farm to fork”), where phages can be applied to ensure dairy safety.

As we mentioned previously, foodborne diseases continue to be a hurdle for human health and those associated to dairy industries are not an exception. Thus, many pathogenic bacteria can spread along the food chain from “farm to fork”. In this regard, phages can be used as antimicrobials and biocontrol agents in food industries to prevent and control step by step the pathogenic bacterial contamination during food production (Figure 3). The use of phages has some advantages over conventional disinfectants such as their narrow host range, targeting specifically bacteria from one species or genus, being also effective against bacteria resistant to antibiotics. Moreover, phages have been described as safe for humans, animals, plants and the environment [79]. Besides, they do not cause equipment or surface damage or alter the organoleptic properties of food.

The efficacy of phages as an intervention strategy in primary production to reduce bacterial infections in food-producing animals has been widely demonstrated [80]. Nevertheless, data regarding the use of phages in the dairy industry are still scarce. The treatment of subclinical *S. aureus* mastitis in lactating dairy cattle with phage K resulted in a cure rate of 16.7%, although the difference between the treated and non-treated groups was not statistically significant. This can be the consequence of phage inactivation in the udder due to milk proteins and fats [81]. However, utilization of phages as biocontrol agents in milk seems to be a better approach, since the combination of two temperate phages ΦH5 and ΦA72 inhibited the growth of *S. aureus* at 37 °C in ultra-high-temperature (UHT) and traditionally pasteurized whole-fat milk [82]. Moreover, lytic derivatives of these phages, Φ88 and Φ35 were successfully used to completely remove *S. aureus* during curd manufacturing and also during the maturation of fresh and hard-type cheeses [83,84]. Similarly, the application of listeriaphages in combination with a bacteriocin (coagulin C23) to extended shelf life (ESL) milk contaminated with *L. monocytogenes* prevented bacterial growth at 4 °C after 10 days [85].

In the dairy industry, recurrent contamination comes from inadequate cleaning of the equipment and the growth of pathogenic bacteria forming biofilms. Biofilms are structures where bacterial cells are protected by a surrounding matrix, thus becoming difficult to clean and remove. Several studies using biofilms preformed in laboratory conditions (onto polystyrene) have confirmed the potential of phages for staphylococcal biofilm removal. Phage K and a mixture of derivative phages removed biofilms in a time-dependent manner, with the highest reduction occurring after 72 h at 37 °C [86]. The combination of phage K with another staphylococcal phage (DRA88), completely removed biofilms after 48 h at 37 °C [87]. In a similar way, phages phiIPLA-RODI, phiIPLA-C1C, and a mixture of both phages, achieved a reduction of 2 log units after 8 h of treatment at 37 °C [88]. On the other hand, *E. coli* biofilms formed onto materials typically used in food processing surfaces (stainless steel, ceramic tile and high density polyethylene) were removed below the detection level after treatment with a phage mixture named BEC8 [89]. Biofilms formed by *L. monocytogenes* onto stainless steel were reduced up to 5.4 log-units/cm^2^ by phage P100 [90]. In this regard, a commercial phage-based product ListShield^TM^, developed by Intralytix Inc. (Baltimore, MD, USA), has been proposed as a disinfectant for food facilities and also on cheese surfaces [91].

The potential of phages in the food industry is so extensive that several companies have developed phage-based products against important foodborne pathogens that could be used as disinfectants and as food-processing aids. But only Intralytix Inc. (Baltimore, MD, USA) and Micreos BV (Wageningen, The Netherlands) commercialize phage-based products (ListShield^TM^ and PhageGuard Listex, respectively) that can be applied in dairy settings. PhageGuard Listex can be applied as a surface intervention against *Listeria* contamination on cheese by spraying or by immersion, without affecting the color, texture or taste of the product [92]. These phage-based products provide a basis for the future approval of phages as disinfectants and preservatives, overcoming the specific regulatory shortcomings of each country.

### 4.2. Regulatory Framework for the Application of Phage-Derived Products in the Food Industry

One of the major difficulties for the use of phages as antimicrobial agents is the lack of a proper regulatory framework for their authorization. Moreover, the European Food Safety Authority (EFSA) expressed concerns regarding the efficacy of phages and the danger of recontamination of the food products [93]. In the case of the dairy industry, and food industry at large, phages have great potential for the control of foodborne pathogens. As mentioned previously, phages can be used as food preservatives or for the disinfection of food-contact surfaces, especially against biofilms. However, depending on their intended use and label claims, the procedure for their approval may vary and, in some cases, be time-consuming and costly. Moreover, legislation can differ considerably from country to country.

Probably the easiest route for placing a phage-based product on the market is for application as a food-processing aid. Indeed, several products have been granted clean label processing in the USA, Canada, Israel, Australia, New Zealand, Switzerland, Norway and the EU (The Netherlands). The first product to be approved by the Food and Drug Administration (FDA) and the US Department of Agriculture (USDA) was LISTEX^TM^ P100, now named PhageGuard Listex, in 2007 (EBI Food Safety, Wageningen, The Netherlands). More recently, three phage-based products manufactured by Intralytix Inc. (Baltimore, MD, USA), have also been approved by the FDA for application in food-processing facilities against *L. monocytogenes*, *E. coli* O157:H7, and several *Salmonella* species.

Another potential application of phage-based products is as food additives. So far, only Intralytics has achieved FDA approval for commercializing the phage product ListShield^TM^ as a food additive.

The approval of phage-based products as surface disinfectants for the food industry is proving to be more complicated than the previously discussed applications. Indeed, only one product, ListShield^TM^, produced by Intralytix Inc., has been granted approval by the FDA and the Environmental Protection Agency (EPA) in the United States to be used for disinfection of non-food contact surfaces and equipment in food-processing facilities and food establishments. In the EU, use of these products as disinfectants in food environments requires authorization under the current Biocidal Products Regulation 528/12 [94]. Preparation of a dossier for this purpose can be somewhat complicated and, most especially, very expensive as it requires a number of studies demonstrating the safety for humans and the environment as well as the efficacy of the active substance, in this case phages, and the product itself. Analysis of potential resistance development is also quite frequently requested by the authorities. 

Overall, despite the obvious difficulties encountered for marketing phage-based products, the need for alternatives to conventional antimicrobials and disinfectants seems to be encouraging progress in this field. Hopefully, this will only be the first step towards the development of a proper legal framework that allows an easier path to authorization and commercialization of phage-based products.

## 5. Concluding Remarks

One century after phages were first described, there is no doubt regarding their importance in diverse fields including ecology, biotechnology, medicine and industrial activities. The dairy industry provides a perfect example of the diverse ways in which bacterial viruses can affect human activities. This review intends to compile these different aspects, both positive and negative, and gives an overview of how phages have in some ways shaped the development of a whole industrial sector. Thus, achieving a good understanding of phages that infect lactic acid bacteria has enabled the development and implementation of strategies to limit the economic losses associated to fermentation failures. On the other hand, phages appear as a viable alternative to conventional disinfectants for application in food industrial surfaces and dairy products themselves. In the midst of a crisis of rising resistance rates to antimicrobials, phages are giving new hope in the fight against bacterial pathogens. Nevertheless, it is still necessary to conduct further research and develop the appropriate regulatory framework in order to ensure that phage disinfection procedures are effective, safe and easily available.
